# Modulation of the p75NTR during Adolescent Alcohol Exposure Prevents Cholinergic Neuronal Atrophy and Associated Acetylcholine Activity and Behavioral Dysfunction

**DOI:** 10.3390/ijms25115792

**Published:** 2024-05-26

**Authors:** Brian T. Kipp, Lisa M. Savage

**Affiliations:** Department of Psychology, Binghamton University-State University of New York, Binghamton, NY 13902, USA; brian.kipp@mssm.edu

**Keywords:** adolescence, alcohol, acetylcholine, neurotrophin, frontal cortex, basal forebrain

## Abstract

Binge alcohol consumption during adolescence can produce lasting deficits in learning and memory while also increasing the susceptibility to substance use disorders. The adolescent intermittent ethanol (AIE) rodent model mimics human adolescent binge drinking and has identified the nucleus basalis magnocellularis (NbM) as a key site of pathology. The NbM is a critical regulator of prefrontal cortical (PFC) cholinergic function and attention. The cholinergic phenotype is controlled pro/mature neurotrophin receptor activation. We sought to determine if p75NTR activity contributes to the loss of cholinergic phenotype in AIE by using a p75NTR modulator (LM11A-31) to inhibit prodegenerative signaling during ethanol exposure. Male and female rats underwent 5 g/kg ethanol (AIE) or water (CON) exposure following 2-day-on 2-day-off cycles from postnatal day 25–57. A subset of these groups also received a protective dose of LM11A-31 (50 mg/kg) during adolescence. Rats were trained on a sustained attention task (SAT) and behaviorally relevant acetylcholine (ACh) activity was recorded in the PFC with a fluorescent indicator (AChGRAB 3.0). AIE produced learning deficits on the SAT, which were spared with LM11A-31. In addition, PFC ACh activity was blunted by AIE, which LM11A-31 corrected. Investigation of NbM ChAT+ and TrkA+ neuronal expression found that AIE led to a reduction of ChAT+TrkA+ neurons, which again LM11A-31 protected. Taken together, these findings demonstrate the p75NTR activity during AIE treatment is a key regulator of cholinergic degeneration.

## 1. Introduction

Binge ethanol consumption during adolescence produces long-lasting impairments in frontocortical-dependent cognition and executive function [1,2,3]. The observed executive dysfunction following adolescent binge drinking may be a function of underlying deficits in attention. However, there have been limited investigations into the lasting effects that binge ethanol exposure dsuring adolescence has on sustained attention performance. Attentional performance is critically modulated by cortically projecting forebrain cholinergic neurons [4,5]. NbM cholinergic neurons from the basal forebrain send projections that terminate within the mPFC, orbitofrontal cortex (OFC), and anterior cingulate cortex. A selective cholinergic lesion of the NbM using the neurotoxin saponin produced impairments in sustained attention, which was restored through administration of an M1 positive allosteric modulation [6]. Furthermore, optogenetic stimulation of cholinergic neurons within the NbM during cued trials of a sustained attention task (SAT) increased the number of correct responses, while stimulation during non-cued trials increased incorrect responses, and inhibition during cued trials increased the number of omissions [7]. Moreover, increases in acetylcholine (ACh) within the PFC during a cued-appetitive response task occur during cue detection [8]. These studies suggest that NbM cholinergic neurons drive the detection of environmental cues, and reductions in cortical ACh release contribute to impairments in attentional performance.

The cholinergic basal forebrain, particularly the NbM, actively participates in frontocortical-dependent tasks including in working memory, attentional processes, and response to reward and punishment [5,7,9,10,11,12,13,14,15]. In addition, recent work has identified an increase in NbM cholinergic activity during both decision making and reward approach in rats on a radial arm maze task [16]. NbM cholinergic neurons respond transiently to both reward and punishment, and the magnitude of response appears to mirror the degree of uncertainty [12,17]. Together, these suggest that basal forebrain cholinergic neurons participate in multiple aspects of cognition, ranging from attention, learning, memory, as well as outcome valence. Thus, reductions in this neuronal population produce cognitive impairments across several cognitive domains. While we are beginning to understand the role the NbM plays in mediating behavior, it is uncertain whether developmental ethanol exposure influences cholinergic signaling dynamics during complex cognitive tasks.

Adolescent intermittent ethanol exposure (AIE) is used to model extreme binge drinking starting early in adolescence and consistently leads to a persistent suppression of choline acetyltransferase (ChAT) expression [18,19,20,21,22]. The AIE-induced suppression of the cholinergic phenotype in the basal forebrain leads to blunted ACh efflux within the frontal cortex and cognitive impairments in both orbitofrontal cortical-dependent reversal learning, as well as medial prefrontal cortical-dependent cognitive flexibility [18,20,23,24] Although we have previously detected a significant reduction in PFC and OFC ACh efflux through in vivo microdialysis during a spontaneous alternation task, the temporal resolution of this method is limited to several minutes and therefore is not appropriate for analysis of discrete ACh signaling dynamics to microbehaviors (choice decision, reward approach, cue detection, etc.). The recent development of an ACh sensor (GRABACh 3.0) is an ideal match for assessments during cognitive testing as it permits the measurement of ACh activity on the order of seconds with in vivo fiber photometry [25,26,27].

The neuropathological mechanisms that lead to the suppression of ChAT+ within basal forebrain neurons following AIE remain elusive. A growing body of literature implicates that the pan-neurotrophin receptor (p75NTR) mediates the neurodegenerative response in cholinergic neurons. The p75NTR has a greater affinity for proneurotrophins (proNGF and proBDNF) compared to mature neurotrophins (NGF and BDNF) [28]. Although p75NTR lacks an internal catalytic domain, it forms receptor complexes with tropomyosin receptor kinases (e.g., TrkA, TrkB) or other adaptor proteins, such as NOGO-66 and Sortilin [29,30,31,32]. The downstream activity of p75NTR signaling is dependent on these receptor complexes, ranging from apoptosis through c-JUN N-Terminal Kinase (JNK) cascade, or facilitation of cell survival through nuclear factor kappa B (NF-ΚB) pathways [31,33,34,35]. In Alzheimer’s disease (AD), the loss of cholinergic neurons is related to reductions in the ratio of Trk receptors to p75NTR [36,37]. LM11A-31, a p75NTR modulator that prevents proneurotrophin-p75NTR signaling [38,39,40,41,42,43], has been found to be effective in treating age-related cholinergic neurodegeneration [41,43,44]. This unique small molecule has structural and chemical features like the NGF loop 1 domain, which is known to interact with p75NTR. LM11A-31 was found to selectively compete with NGF and proNGF binding to p75NTR—but not to TrkA [39]. It was found to promote survival signaling through p75NTR-dependent mechanisms but also inhibited proneurotrophin-mediated cell death [39]. In addition, LM11A-31 not only prevented AD-associated cholinergic pathology, but it also reversed cholinergic atrophy when treatment began during late-stage amyloid beta deposition [39,41,42,44]. Thus, LM11A-31 may shed light on the role of the p75NTR in developmental alcohol-related neuropathology produced by AIE. Although there are limited examinations in the use of LM11A-31 during developmental timepoints, or with ethanol, this compound has been administered to Sprague Dawley rats at postnatal day 35, daily for two weeks, without gross abnormalities [45] and has previously been used in an adult ethanol model to attenuate excessive alcohol consumption [38].

The goal of the proposed study is twofold: Firstly, the goal is to investigate discrete AIE-associated deficits in cortical cholinergic activity during a sustained attention task. Secondly, we aim to elucidate whether the p75NTR mediates the loss of cholinergic phenotype and, more importantly, if modulating the p75NTR during developmental ethanol exposure conserves the cholinergic phenotype and protects cognitive performance in adulthood.

## 2. Results

### 2.1. Statistical Analyses

For the measurement of change in body weight over the course of treatment, a repeated measures ANOVA was used with between-subjects factors of treatment condition (AIE and CON) and drug (LM11A-31 and vehicle); the within-subject factor was day of gavage. Male and female analyses for change in body weight over the course of treatment were run separately. BECs were measured using a 2-way ANOVA (drug, sex), and AIE and CON were run separately, such that the analysis reflects AIE comparison to AIE-LM and CON comparison to CONLM. Operant data for the pSAT and SAT tasks prior to and post-surgery were run using a 3-way ANOVA (treatment, drug, and sex) with Fisher’s LSD post hoc analyses used to examine group differences when the omnibus F was significant. Since analysis of the fiber photometry data revealed no significant main effects of sex in the 3-way ANOVA (all *p*s > 0.05), sex as a factor was collapsed, and data were analyzed as a 2-way ANOVA with Fisher’s LSD post hoc analyses. Lastly, NbM ChAT+TrkA+ and ChAT+TrkA- data were analyzed using a 3-way ANOVA (sex, treatment, and drug) with Fisher’s LSD post hoc analysis for group differences. All statistical analyses were conducted in prism 9.

### 2.2. Treatment Growth Curves and BECs

#### 2.2.1. Male Growth Curve

**Figure 1:**
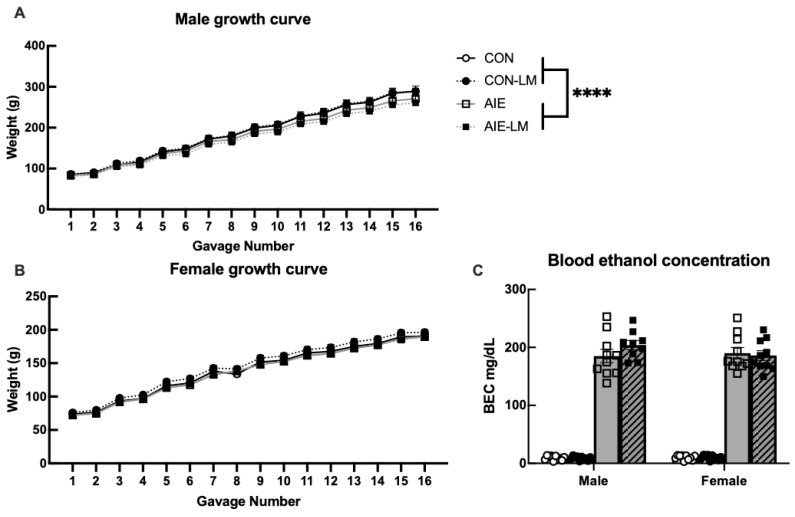
**Growth curves and blood ethanol concentrations**. Graphs depicting the change in body weight of male and female Sprague Dawley rats over the course of AIE or CON treatment with or without LM11A-31 administration. All subjects significantly increased weight gain over the course of treatment. In males, CON-V and CON-LM males gained more weight than AIE-V and AIE-LM males (**A**). Similarly, female weight gain over the course of treatment did not significantly differ across CON and AIE treatment conditions or with the administration of LM11A-31 (**B**). Blood ethanol concentration measured from tail bloods collected 1 h following the eighth gavage. Solid grey bars are AIE-V and hashed gray bars are AIE-LM. LM11A-31 treatment did not significantly alter BECs in CON- or AIE-treated animals during treatment in adolescence (**C**). Data represent group average ± SEM. **** Indicates *p* < 0.0001.

When analyzing the change in body weight of males over the course of AIE treatment, it was found that Mauchly’s test of sphericity was violated (*p* < 0.001); therefore, a Greenhouse–Geisser correction was applied for within-subject analyses. Over the course of treatment, all males significantly increased in body weight from PND 25–57 (F(1.85, 64.74) = 4453.924, *p* < 0.001; Figure 1A). However, CON water-treated rats had a greater increase in body weight during treatment than AIE males (F(1.85, 64.74) = 11.724, *p* < 0.001). Treatment with LM11A-31 did not influence change in body weight during development (*p* > 0.05).

#### 2.2.2. Female Growth Curve

A Greenhouse–Geisser correction was also applied to within-subject analyses for female growth curve data as Mauchly’s test of sphericity was violated (*p* < 0.001). Similar to males, female weight also significantly increased during treatment from PND 25–57 (F(2.74, 98793) = 2218.26, *p* < 0.0001), and LM11A-31 did not alter the growth trajectory in adolescence (*p* > 0.05). Unlike the males, female weight change was not affected by AIE (*p* > 0.05; Figure 1B).

#### 2.2.3. Blood Ethanol Concentrations

To determine if LM11A-31 affected blood ethanol concentrations (BEC) during AIE treatment, tail bloods were collected following the eighth gavage, revealing that CON-LM- did not differ from CON-V-treated rats (*p* = 0.97) nor were sex differences present (*p* = 0.38; Figure 1C). The comparison of AIE-LM and AIE-V animals revealed that LM11A-31 did not affect BEC during AIE treatment (*p* = 0.44), and no differences were observed between males and females (*p* = 0.50). In both cases, no significant sex or sex × drug interactions were detected (*p*s > 0.05).

### 2.3. SAT Pretraining (pSAT)

The number of sessions required to pass the pSAT task was investigated for group differences, revealing a significant main effect of sex (F(1, 36) = 12.4, *p* = 0.0012), where on average, females (16.13, SEM = 1.21) took significantly more sessions to reach criterion than males (11.64, SEM = 0.94). Since males and females differed significantly in the number of sessions to pass pSAT, separate analyses were conducted to determine if treatment and/or drug effects were present in males and females. In both males and females, the number of sessions required to pass pSAT was unaffected by treatment, drug, or the interaction of the two factors (all *p*s > 0.15).

**Figure 2:**
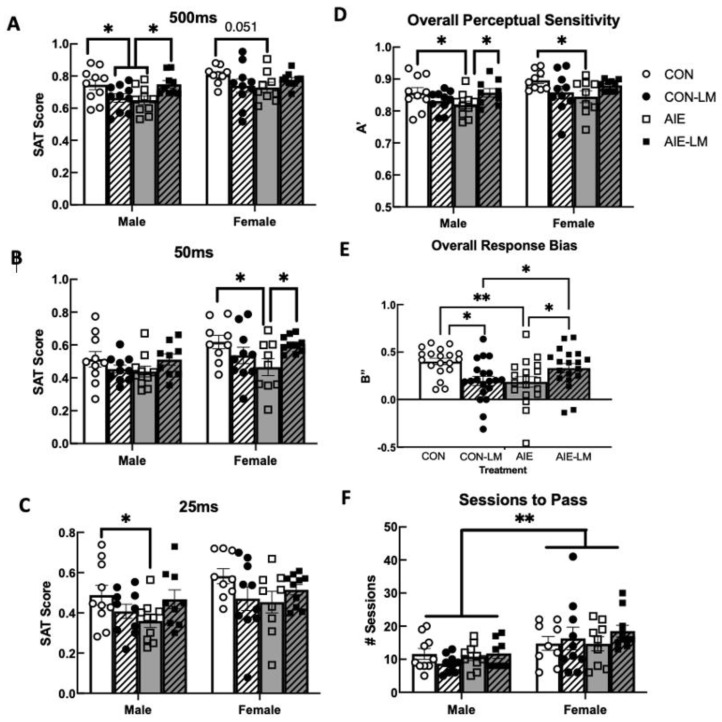
**Performance during pretraining of the sustained attention task**. SAT score and signal detection theory measures on the SAT task in the absence of the house light (pSAT). Data represent group mean ± SEM. AIE-V had lower SAT scores, which were recovered in AIE-LM males. However, LM11-31A treatment in CON male rats had the opposite effect; it impaired performance. AIE-V females performed worse than CON-V females on 500 ms trials, an effect not seen in rats that received LM11-31A (**A**). Under the 50 ms cue duration, AIE-V females performed significantly worse than CON-V and AIE-LM females; however, no group differences were found in male rats (**B**). At the 25 ms cue duration, only male AIE-V rats performed worse than their CON-V counterparts, an effect not seen in LM11-31A-treated rats (**C**). Measures of perceptual sensitivity in AIE-V-treated male and female rats were lower than male and female CON-V rats, which was not seen when LM11-31A was given (**D**). Regardless of sex, AIE-V- and CON-LM-treated rats had a more liberal response bias compared to CON-V and AIE-LM rats (**E**). Lastly, females overall took significantly more sessions to complete pSAT than males, regardless of treatment (**F**). * Indicates *p* < 0.05; ** indicates *p* < 0.01.

To determine if cue duration significantly impacted performance on the pSAT task, a within-subjects ANOVA was conducted on SAT scores across the 500 ms, 50 ms, and 25 ms cue durations (see Figure 2A–C). Sphericity was found to have been violated (*p* < 0.001); therefore a Greenhouse–Geisser correction was applied. As the cue duration shortened, SAT scores were reduced regardless of treatment (F(1.56, 98.43) = 374.97, *p* < 0.0001). Group differences were also investigated during the pSAT task at each cue duration to identify underlying group differences in attentional performance. When examining the 500 ms cue duration, a significant main effect of sex was observed (F(1, 36) = 8.47, *p* = 0.006), where females performed significantly better than males (0.70, SEM = 0.01). Therefore, males and females were run in separate analyses. In males, a significant treatment x drug interaction was detected (F(1, 35) = 11.1, *p* = 0.002). Post hoc analyses revealed that male CON-V rats had higher SAT scores compared to AIE-V male rats (*p* = 0.015) and CON-LM males (*p* = 0.03). Additionally, male AIE-LM-treated animals performed better at the 500 ms cue duration that AIE-V-treated animals (*p* = 0.015). When examining pSAT performance in females, a trending treatment x drug interaction was observed (F(1, 34) = 3.99, *p* = 0.053). Post hoc tests revealed a trending difference, where female AIE rats had lower SAT scores than CON females.

Similar to the 500 ms cue duration, females had significantly higher SAT scores at the 50 ms cue duration than males (F(1, 36) = 7.484, *p* = 0.0096); therefore, males and females were run in separate analyses. At the 50 ms cue duration in males, no treatment (*p* = 0.86), drug (*p* = 0.81), or treatment x drug interactions were observed (*p* = 0.07). However in females, a significant treatment x drug interaction was detected (F(1, 33) = 11.03; *p* = 0.012). Post hoc tests revealed that AIE-V-treated females had lower SAT scores compared to CON females and AIE-LM-treated females (*p* = 0.025). 

Under the 25 ms cue duration, females outperformed males on the pSAT task (F(1, 36) = 4.88; *p* = 0.033). Since males and females differed significantly at the 25 ms cue duration, males and females were run separately. In males, at the 25 ms cue duration, a significant treatment x drug interaction was detected (F(1, 35) = 5.22; *p* = 0.028). Post hoc tests determined that male CON rats had higher SAT scores compared to male AIE rats (*p* = 0.031). In females, no significant differences were observed across treatment conditions (*p* = 0.36) or drug (*p* = 0.58), and no treatment x drug interaction was found (F(1, 34) = 3.57, *p* = 0.067).

Measures of perceptual sensitivity and response bias allow for examination of attentional performance without the influence of the behavioral strategy that the rats used (see Figure 4C–E). It was discovered that males and females significantly differed in perceptual sensitivity (F(1, 36) = 8.357, *p* = 0.0095), where females had higher perceptual sensitivity scores compared to males. Since males and females differed significantly in A′, males and females were separated prior to subsequent analyses of treatment of drug effects. In males, a significant treatment x drug effect was observed (F(1, 35) = 7.73, *p* = 0.0087). Post hoc analyses revealed that male CON- and AIE-LM-treated (*p* = 0.048) rats had significantly higher A’ scores compared to AIE-V-treated males. In females, a significant treatment x drug interaction was detected (F(1, 34) = 5.17; *p* = 0.029). Further post hoc tests determined that female CON rats scored higher on perceptual sensitivity than AIE-treated females (*p* = 0.021). 

Regarding response bias, a main effect of sex was not detected (*p* = 0.065); therefore, sex was collapsed across groups for further analyses. A significant treatment x drug interaction was detected (F(1, 73) = 13.59, *p* = 0.0004). Post hoc analyses revealed that CON-V-treated animals utilized a more conservative response strategy compared to CON-LM-treated animals (*p* = 0.03) and AIE-V-treated rats (*p* = 0.002). In addition, AIE-LM rats demonstrated a more conservative response strategy than AIE-V-treated rats (*p* = 0.03) and CON-LM-treated rats (*p* = 0.045).

Performance on pSAT was examined for group differences in the latency to lever press and latency to collect reward. Neither treatment, drug, nor their interaction were found to have affected latency to lever press on pSAT. Males and females did not differ significantly in the latency to lever press on 500 ms trials (*p* = 0.10). For the 50 ms cue duration, a significant main effect of sex was detected (F(1, 68) = 6.54, *p* = 0.012), where females (370 ms, SEM = 26.2) were significantly slower to lever press compared to males (310 ms, SEM = 10.1). Similarly, a significant main effect of sex was also detected at the 25 ms cue duration (F(1, 68) = 6.56, *p* = 0.012), where females (370 ms, SEM = 27.8) were significantly slower to respond to lever presentation compared to males (310 ms, SEM = 11.0). During non-cue trials, latency to lever press was unaffected by any variable (all *p*s > 0.22).

Latency to collect reward was also measured following correct response to trials. On 500 ms trials, a significant treatment x sex interaction was present (F(1, 30) = 5.01; *p* = 0.03); female CON-V and CON-LM females (390 ms, SEM = 36.2) collected the reward more quickly than CON-V and CON-LM males (580 ms, SEM = 129.9). In males, latency to collect reward was unaffected by AIE, LM11A-31, or the interaction (all *p*s > 0.15). Similarly in females, neither treatment, drug, nor the treatment x drug interaction (all *p*s > 0.50) affected latency to collect reward. At the 50 ms cue duration, a sex x treatment interaction was found (F(1, 31) = 7.54; *p* = 0.01). Again, female CON-V rats (392 ms, SEM = 35.9) were faster to collect the food reward than CON-V and CON-LM males (485 ms, SEM = 62.7). At the 25 ms cue duration, no factors affected latency to collect reward (all *p*s > 0.20). Lastly, latencies to collect reward during non-cue trials were examined for differences across groups. Males and females significantly differed in reward latencies on non-cue trials (F(1,66) = 5.93; *p* = 0.017): Females (370.4 ms, SEM = 28.7) were quicker to collect the food reward at the end of correct trials than males (521.9 ms, SEM = 61.8). No other factors affected reward collection latencies.

### 2.4. SAT Task

**Figure 3:**
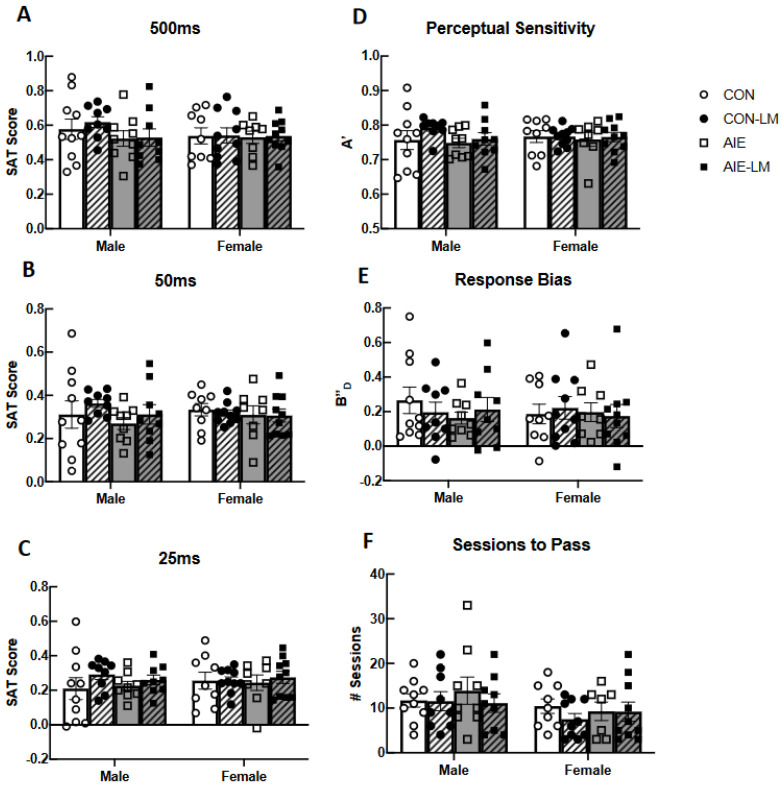
**Performance on the sustained attention task**. SAT performance and signal detection theory measures during the house light-on condition. Data represent group means ± SEM. In both male and female rats, no significant group differences were found in SAT scores across the 500 ms, 50 ms, and 25 ms cue durations (**A**–**C**). Groups did not differ in signal detection theory measures of perceptual sensitivity or response bias, nor were there differences in the number of sessions required to complete the SAT task (**D**–**F**).

SAT scores were calculated following completion of the SAT task and analyzed as a function of cue duration (See Figure 3A–C). Again, a repeated measures ANOVA was used to determine if performance on the SAT declined with shorter cue durations. Similar to pSAT, sphericity was found to have been violated and after applying a Greenhouse–Geisser correction, SAT performance significantly declined as cue duration decreased (F(1,53) = 284.91, *p* < 0.0001). For the 500 ms, 50 ms, and 25 ms cue duration, no significant main effects of treatment, drug, or sex were detected, and no significant interactions were observed (*p*s > 0.05). Similarly, no significant differences were detected on measures of perceptual sensitivity, response bias, or the number of sessions required to master the task (all *p*s > 0.05; Figure 3D–F).

Latencies to lever press and reward were also examined. While no significant differences in latency to lever press were detected in the 500 ms, 50 ms, 25 ms, and the non-cued trials (all *p*s > 0.05), specific groups did not differ significantly in the latency to collect reward (all *p*s > 0.05).

### 2.5. GRAB ACh 3.0 Activity in the mPFC during SAT

**Figure 4:**
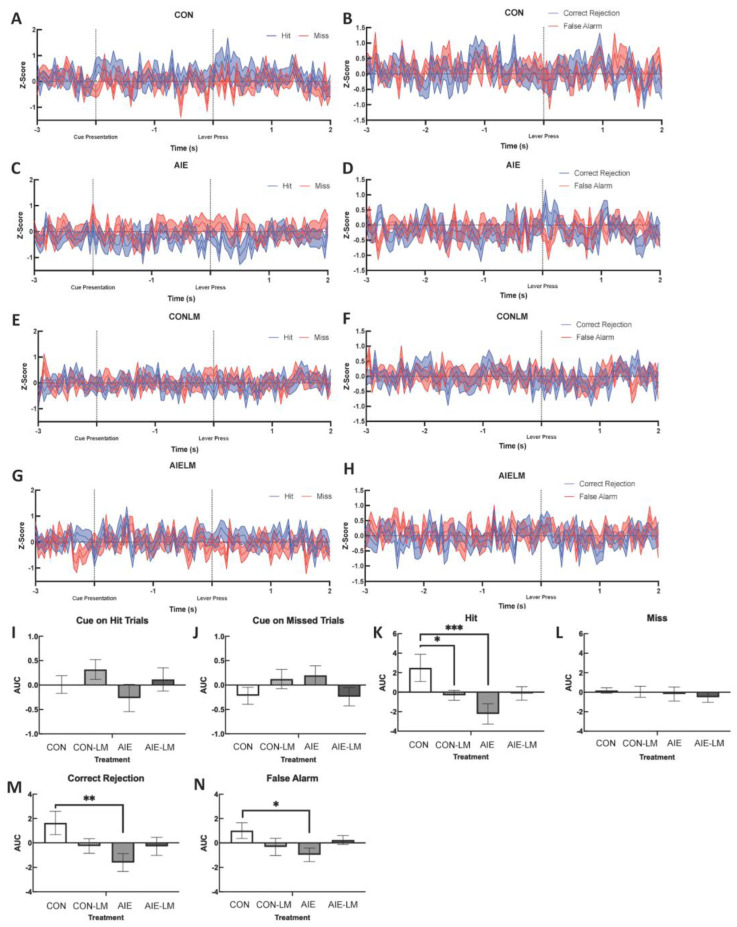
**Activity of GRAB ACh 3.0 during SAT and area under the curve (AUC) measures**. Example of a plot of group-averaged Grab ACh 3.0 activity recorded via fiber photometry during signal (**A**,**C**,**E**,**G**) and non-signal trials (**B**,**D**,**F**,**H**) of SAT. Data represent Z-score transformation of signal relative to baseline recording during the previous intertrial interval. Area-under-the-curve measurements during cue presentation, response selection, and the post-reinforcement period during the SAT task (**I**–**N**). Data represent- the AUC ± SEM. No group differences were observed during the cue presentation on hit and miss trials (**I**,**J**). However, CON-V rats had a greater AUC during the post-response period following hits compared to AIE-V- and CON-LM-treated animals. LM11-31A treatment prevented this deficit (**K**). No group differences were present in the AUC during misses (**L**). CON rats also had greater AUC compared to AIE rats in the post-response period following correct rejections (**M**) and false alarms (**N**), which were not corrected by LM11-31A treatment in AIE-treated rats. * Indicates *p* < 0.05; ** *p* < 0.01, *** *p* < 0.005.

ACh signaling was measured through in vivo fiber photometry during the sustained attention task; trials were broken down to record responses to cue presentation, as well as response to reward (See Figure 4A–H for group averaged recordings). Area-under-the curve measurements of ACh activity in the mPFC did not reveal significant differences during cue presentation of trials of hits or misses (all *p*s > 0.05; Figure 4I,J). Following hits, a significant main effect of treatment was observed (F(1, 61) = 5.96, *p* = 0.017), where AIE-treated rats had a lower area under the curve (AUC) than CON rats (Figure 4K). In addition, a treatment x drug interaction was also observed following hits (F(1, 61) = 7.04, *p* = 0.01). Post hoc testing revealed that CON-V rats had a greater AUC than CON-LM- (*p* = 0.036) and AIE-V-treated rats but not AIE-LM rats. Significant differences in AUC during misses were not observed across treatment or drug exposure (all *p*s > 0.05, Figure 4L). Following correct rejections during non-signal trials, a main effect of treatment was observed (F(1, 61) = 4.74, *p* = 0.033), where AIE-treated rats had a lower AUC compared to CON rats (Figure 4M). A significant treatment x drug interaction was also detected during correct rejections (F(1, 61) = 4.58, *p* = 0.036). Again, post hoc testing revealed that AIE-treated rats had a lower AUC following the lever press than CON rats. Lastly, during false alarm responses on non-signal trials, a significant treatment x drug interaction was observed (F(1, 61) = 4.59, *p* = 0.036). Post hoc tests revealed that AIE-treated rats had a lower AUC during the post-response period compared to CON rats (*p* = 0.024, Figure 4N).

**Figure 5:**
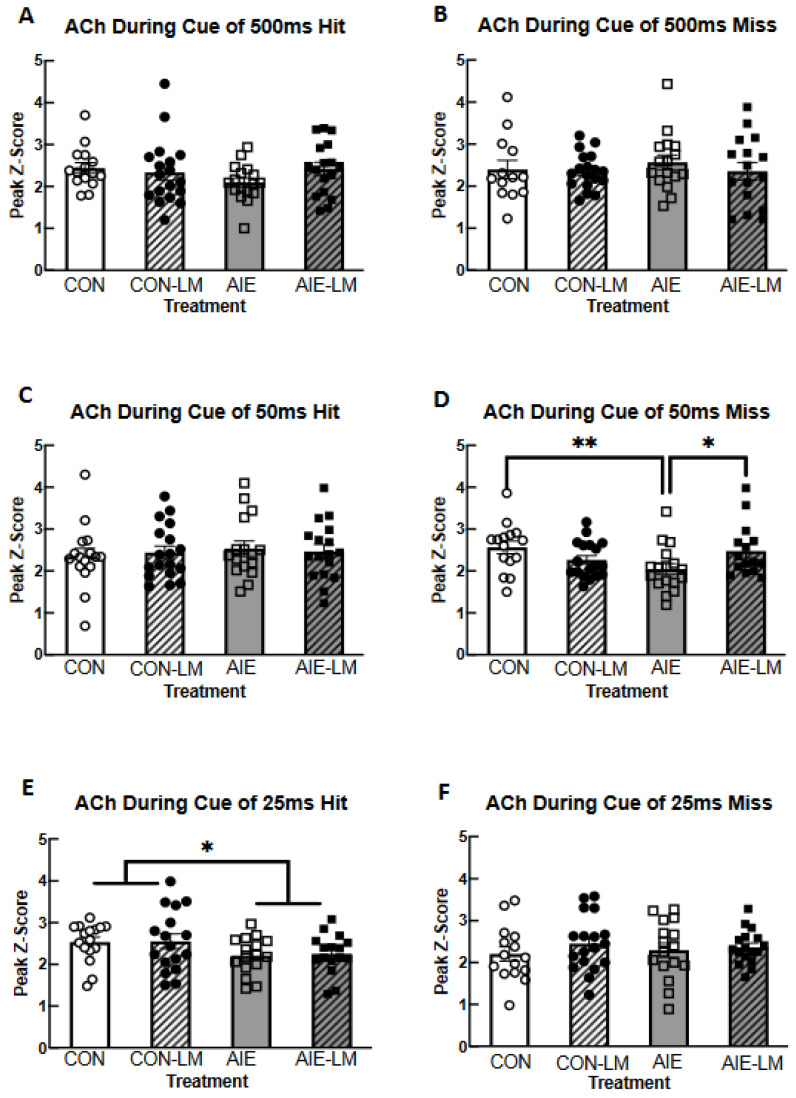
**Neural activity assessed by GRAB ACh 3.0 during cue presentation**. Grab ACh 3.0 recording during cue presentation on the SAT task. Data represent mean peak Z-score ± SEM. No group differences were found in ACh 3.0 activity during cue presentation on correct 500 ms, 50 ms, or 25 ms cue duration trials (**A**,**C**,**E**). No group differences were found in peak z-score during cue presentation of 500 ms cue miss trials (**B**). However, during 50 ms cue presentation of miss trials, AIE-V-treated animals had a significantly lower peak z-score relative to CON-V- and AIE-LM-treated animals (**D**). No group differences were observed on the 25 ms cue trials that resulted in a miss (**F**). * Indicates *p* < 0.05; ** *p* < 0.01.

When examining ACh signaling during cue presentation, it was found that groups did not differ in peak Z-score recorded at the 500 ms cue duration in trials that would ultimately become hits (all *p*s > 0.05; Figure 5A) or misses (all *p*s > 0.05; Figure 5B). The peak z-score in response to the 50 ms cue during trials that would become hits did not differ across treatment or drug conditions (all *p*s > 0.05; Figure 5C). During cue presentation of 50 ms miss trials, a significant treatment x drug interaction was identified (F(1, 61) = 7.21, *p* = 0.0093; Figure 5D). During 50 ms cue trials, ACh activity was significantly lower in AIE-V-treated male and females compared to CON-V (*p* = 0.0098) and AIE-LM (*p* = 0.029) conditions. During 25 ms trials, a significant treatment effect was observed on hit trials (F(1, 61) = 5.23, *p* = 0.025; Figure 5E) but not miss trials (*p*s = 0.98; Figure 5F): CON-V and CON-LM treatment conditions had greater peak z-scores than AIE-V and AIE-LM treatment conditions. Main effects of drug were not found on 25 ms hit trials (*p* = 0.82) or miss trials (*p* = 0.31), nor were there interaction effects on hit trials (*p* = 0.92) or miss trials (*p* = 0.56).

**Figure 6:**
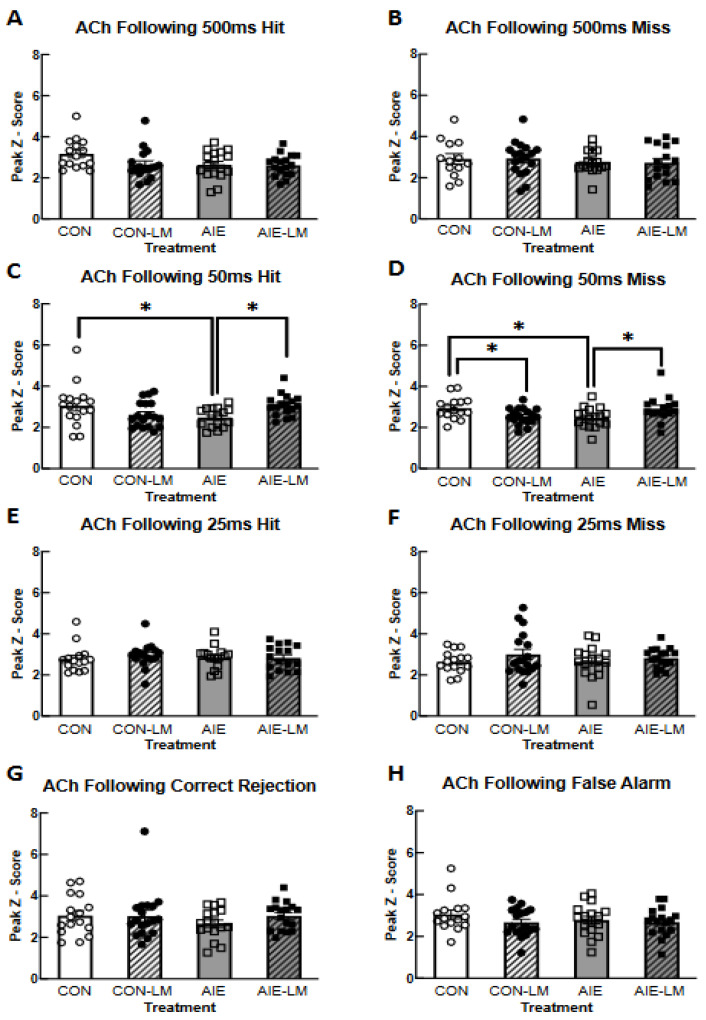
**Activity of GRAB ACh 3.0 in the prefrontal cortex during hits, misses, correct rejections, and false alarms**. Grab ACh 3.0 activity measured with fiber photometry during the post-response period of correct and incorrect trials (**A**–**F**). Data represent the mean peak z-score ± SEM. While no group differences were evident following 500 ms cue trials (**A**,**B**), the peak z-score of ACh 3.0 activity was significantly lower in AIE-V-treated rats compared to CON-V and AIE-LM rats following hits and misses on 50 ms trials (**C**,**D**). Following a miss on the 50 ms cue trial, peak z-score of GRAB ACh 3.0 was lower in the CON-LM treatment condition compared to CON-V. Groups did not differ in peak z-score following 25 ms hits or misses (**E**,**F**). Groups also did not differ in peak z-score following correct rejections and false alarms of non-signal trials (**G**,**H**). * Indicates *p* < 0.05.

ACh activity was also measured in the form of peak z-score immediately following hits, misses, correct rejections, and false alarms. When examining cued trials, it was found that ACh activity did not differ on 500 ms trials following hits (all *p*s > 0.09; Figure 6A) or misses (all *p*s; Figure 6B). During the reward period of 50 ms hits, a significant treatment x drug interaction was detected (F(1, 63) = 9.34, *p* = 0.0033; Figure 6C). Following correct choice on 50 ms trials, the peak z-score of ACh activity was significantly lower in AIE-V rats when compared to CON-V- (*p* = 0.021) and AIE-LM-treated animals (*p* = 0.017). A significant treatment x drug interaction was also observed following misses on 50 ms trials (F(1, 61) = 10.53, *p* = 0.0019; Figure 6D). The peak z-score of ACh activity in the mPFC of CON-V rats was significantly higher than the AIE-V and CON-LM groups (both *p*s < 0.04). Moreover, the AIE-LM condition had a higher ACh peak z-score than AIE-V (*p* = 0.02) rats. No significant differences were observed in the period of recording following hits and misses on the 25 ms cue duration (all *p*s > 0.05; Figure 6E,F). In addition, no group differences were observed in the peak z-score of ACh activity on the non-trials, following correction rejections (all *p*s > 0.05; Figure 6G) and false alarm trials (all *p*s > 0.05; Figure 6H).

**Figure 7:**
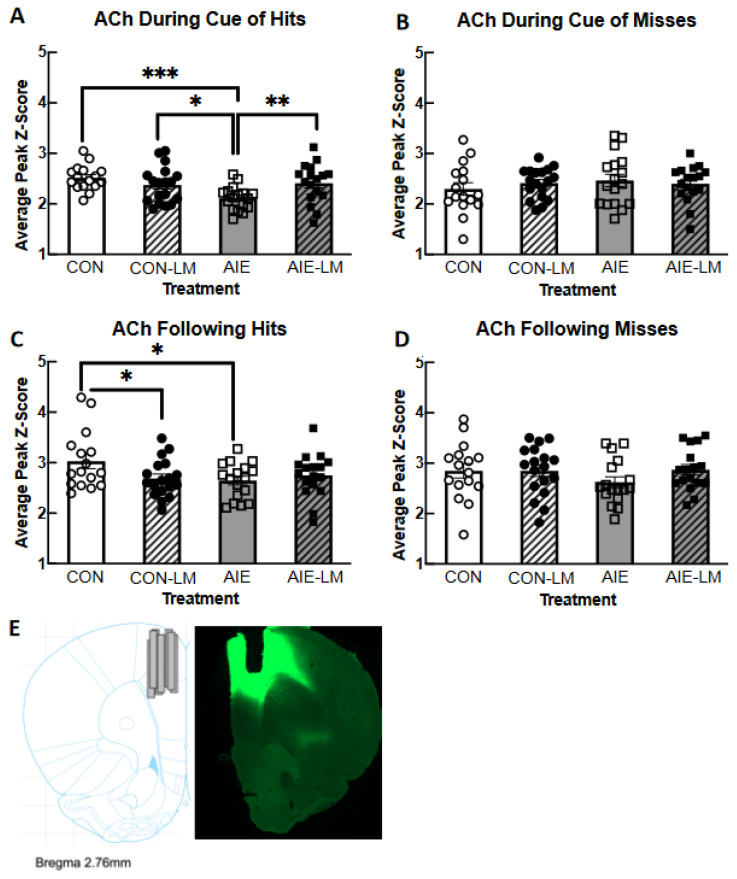
**Activity of GRAB ACh 3.0 collapsed across cue duration**. Average Grab ACh 3.0 activity in the mPFC during cue presentation and the post-response period across all cue durations. Data represent the group mean peak z-score ± SEM. During cue presentation on hit trials, AIE-V-treated animals had significantly lower peak z-score of ACh 3.0 activity compared to CON-V, CON-LM, and AIE-LM treatment conditions (**A**). During the post-response period of hit trials, CON-V-treated animals had higher peak z-score ACh 3.0 activity compared to CON-LM and AIE-V treatment groups (**B**). Group differences in peak z-score were not detected during cue presentation of miss trials (**C**) or during the post-response period of miss trials (**D**). * Indicates *p* < 0.05; ** *p* < 0.01; *** *p* < 0.005. Sample Grab ACh 3.0 viral expression and fiber optic cannula placement in the mPFC (2.70 AP, ±0.7 ML, −3.0 DV); image from Paxinos and Watson (2014) (**E**).

The 500 ms, 50 ms, and 25 ms cue duration trials were averaged together to determine if groups differed in peak z-scores of cued trials in general. Examining the peak z-score of ACh activity during the cue of hits revealed a treatment x drug interaction (F(1, 64) = 8.11, *p* = 0.0059; Figure 7A). Fisher’s LSD post hoc analyses revealed that AIE-V rats had lower peak ACh release than CON-V (*p* = 0.0005), AIE-LM (*p* = 0.0083), and CON-LM (*p* = 0.017) rats. It was also found that the ACh signal significantly differed in the post-response period of hit trials as a function of treatment x drug (F(1, 64) = 4.33; *p* = 0.041; Figure 7C). Fisher’s LSD revealed that CON-V-treated animals had greater peak z-scores than the AIE-LM (*p* = 0.015) and CON-LM (*p* = 0.028) groups. Interestingly, groups did not differ in response to the cue of miss trials or in the post-response period following a miss (all *p*s > 0.05; Figure 7B,D).

### 2.6. NbM ChAT+ TrkA+ Cell Counts

**Figure 8:**
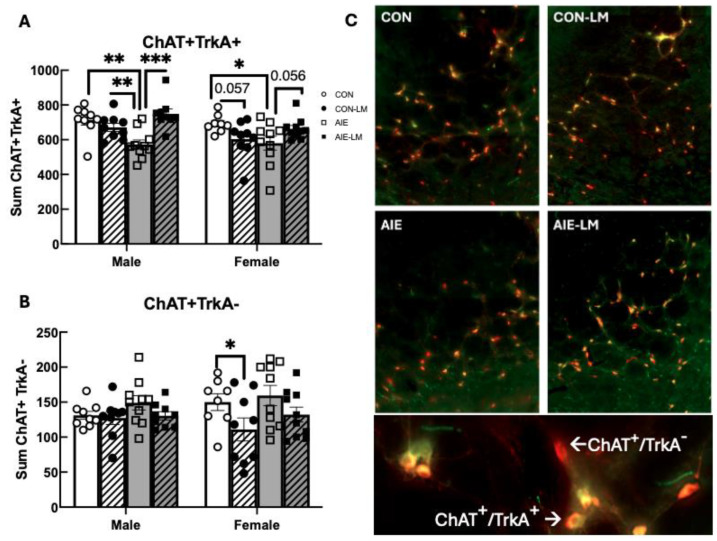
**Cell counts for ChAT+, TrkA+ neurons in the NbM**. The sum number of fluorescently labeled ChAT+ neurons in the NbM and the number of ChAT+TrkA+ and ChAT+TrkA- phenotypes. Data represent group means of the total number of counted cells across four sections ± SEM. Group differences were evident in the ChAT+TrkA+ phenotype of cholinergic neurons in the NbM. In males, AIE-V-treated rats had significantly fewer ChAT+TrkA+ labeled neurons than CON-V, CON-LM, and AIE-LM rats. In females, AIE-V-treated rats also had fewer ChAT+TrkA+ cells than CON-V, CON-LM, and AIE-LM females, while CON-LM-treated rats had fewer ChAT+TrkA+ cells than CON-V females (**A**). Group differences in ChAT+TrkA- cell counts in males were not evident; however, CON-LM females had fewer ChAT+TrkA- cells in the NbM compared to CON-V females (**B**). Sample images of NbM sections for ChAT+TrkA+ (Gold) and ChAT+TrkA- (Red) (**C**). ChAT-TrkA+ cells were not detected in these sections. All images were recorded at 20× magnification. * Indicates *p* < 0.05; ** *p* < 0.01; *** *p* < 0.005.

ChAT-TrkA co-expression was found to be significantly affected by sex as males had more ChAT+TrkA+ cells than females (F(1, 66) = 4.22; *p* = 0.043; Figure 8A). Since males and females differed in ChAT+TrkA+ expression, separate analyses were run for males and females. In males, a treatment x drug interaction was found (F(1, 34) = 17.37, *p* = 0.0002). AIE led to a reduction in the number of ChAT+TrkA+ neurons in the NbM in males compared to CON males (*p* = 0.0006). However, LM11A-31 recovered the ChAT+TrkA+ population in AIE rats (*p* < 0.0001). In females, a significant treatment x drug interaction was also detected (F(1, 33) = 7.82; *p* = 0.008): AIE females had fewer ChAT+TrkA+ cells than CON females (*p* = 0.015). AIE-LM females also had more ChAT+TrkA+ cells in the NbM compared to AIE-V females (*p* = 0.055).

When examining the ChAT+TrkA− phenotype, differences were not observed as a function of treatment (*p* = 0.11) or sex (*p* = 0.65); however, LM11A-31 did have an effect on the number of ChAT+TrkA− cells in the NbM (F(1, 31) = 7.36, *p* = 0.011; Figure 8B), driven by reductions in CON-LM females compared to CON-V females.

## 3. Materials and Methods

### 3.1. Subjects

Eighty male and female Sprague Dawley rats that were bred from dams received from Envigo (Indianapolis, IN, USA) at Binghamton University were used to generate 10 rats per sex per treatment condition. Rats were randomly assigned treatment conditions with no more than 1 subject per sex from each litter. On postnatal day (PND) 21, male and female rats were weaned and double/tripled housed (same sex) in a temperature- and humidity-controlled vivarium under a 12 h light–dark cycle (0700–1900) at Binghamton University. Rats were provided with ad libitum access to food chow (Purina Lab Diet 5012) and water and were provided with environmental enrichment in the form of nesting packets and wooden chew blocks.

### 3.2. Treatment

**Figure 9:**
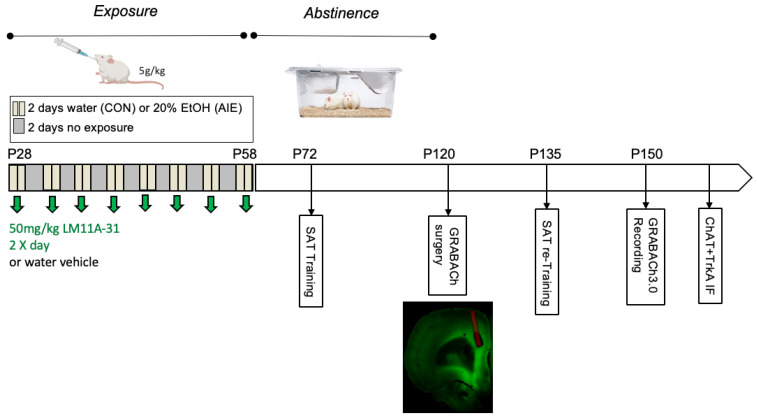
Experiment 2 treatment timeline. Groups underwent the previously described AIE or CON treatments. Separate AIE + LM11A-31 and CON + LM11A-31 treatment groups received IG gavage of 50 mg/kg LM11A-31, 30 min before and 8 h following each gavage. Animals were trained on SAT 2 weeks following the end of AIE treatment. Following mastery of SAT, subjects underwent ACh GRAB 3.0 viral infusion into the mPFC with fiber optic cannula 2 weeks after the end of AIE. Three weeks following viral infusion and SAT re-training, groups underwent operant pretraining and behavioral testing on the SAT, where mPFC ACh activity is recorded through in vivo fiber photometry.

Rats were exposed to one of four treatment conditions (~10 per sex/treatment/drug exposure). A standard model of heavy adolescent ethanol exposure was used [2,18,19,20,21,22,23,24]: AIE-treated rats received intragastric gavages of 5 g/kg 20% EtOH and control-treated rats (CON) received volume-matched equivalents of water to control for gavage effects, following two-day-on two-day-off cycles from PND 25–57 (See Figure 9 for a timeline of treatment and behavioral testing). This timeframe was chosen as it encapsulates both early and late adolescence. The third treatment group, AIE-LM, received the identical ethanol treatment as AIE; however, this group also received 50 mg/kg of the p75NTR modulator LM11A-31 (MedChemExpress, Monmouth Junction, NJ, USA) through intragastric gavage 30 min before and 8 h following each water or EtOH gavage. The last treatment condition, CON-LM, underwent the same treatment schedule as CON-treated animals; however, this group also received 50 mg/kg LM11A-31 30 min before and 8 h following each gavage. In addition to the water and ethanol administered, rats that did not receive LM11A-31 received an additional intubation of water (vehicle; V) to match the volume of LM11A-31 administered. One hour after the 8th gavage, BECs were collected from tail veins of all treatment conditions and were measured (GM7 Analyzer, Analox, London, UK).

### 3.3. Operant Sustained Attention Task

Operant chambers (30 cm × 33 cm × 23 cm; Med Associates Inc., St. Albans, VT, USA) located within sound-attenuating shells (59 cm × 55 cm × 36 cm), interfaced with MED-PC IV (Med Associates Inc.) and Synapse recording software (version 3, Tucker Davis Technology, Alachua, FL, USA) were used for operant training and fiber photometry recording. Each operant chamber contained two retractable levers situated on the left and right of a food trough through which a single food pellet (Rodent Purified Dustless Precision Pellet; Bio-Serve, Flemington, NJ, USA) was dispensed upon correct responses. The operant chambers also contained a single cue light located directly above the food trough and a house light positioned on the opposite wall of the chamber. Behavioral inputs from the operant chamber were time-stamped in Synapse via TTL pulses using the digital input and output ports from ICON4 system (Tucker Davis Technologies) that interfaced directly with the fiber photometry recording system (RZ10x Processor; Tucker Davis Technology). All rats were food restricted to 85% of their baseline free-feed weight over the course of one week and were maintained at this restriction, determined by a growth curve, throughout behavioral testing. In addition, rats were handled in the week leading up to operant training.

Two weeks after the end of AIE treatment, rats underwent pretraining and an operant SAT task following a fixed-ratio-1 (FR-1) schedule. During the pretraining phase, rats were conditioned to lever press the left and right lever for 30 min across two consecutive days. On the third day of pretraining, rats began retractable lever training. Retractable lever training consists of 90 trials with the goal of training rats to respond within 10 s of lever presentation. This training also occurred on an FR-1 schedule. Rats were required to make fewer than 5 trial omissions within 90 trials for two consecutive days.

Following completion of pretraining, rats were trained on the SAT pretraining (pSAT). pSAT is a simple discrimination task composed of 81 cued and 81 non-cued trials (Figure 2). Rats are trained to detect the illumination of a central cue light of varying duration (500 ms, 50 ms, and 25 ms) that occurs 2 s prior to lever presentation. On cued trials, rats were rewarded for pressing the left lever (hit, h), and a right-lever press was not rewarded (miss). Conversely, on non-cued trials, a left-lever press was not rewarded (false alarm, f), whereas right-lever presses were rewarded (correct rejection). pSAT took place without general illumination provided by the house light. Mastery of the task was assumed when rats had with 3 consecutive sessions with >70% hits at the 500 ms cue duration and >70% correct rejections. Following this criterion, rats were moved onto the SAT task that had under general illumination of the house light. The inclusion of the house light requires the rats to be vigilant to the cue [46]. The same task contingencies that occurred under pSAT applied to the SAT. Four female rats were unable to meet the passing criteria of the SAT task and were excluded from all measures (two females from the CON group, one female AIE, and one female AIE-LM).

**Figure 10:**
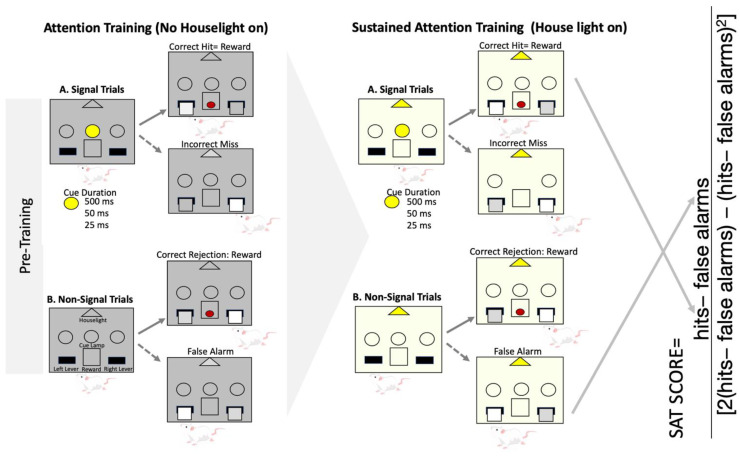
**Sustained attention task and SAT score**. Depiction of the SAT pretraining, SAT tasks, and formula for the development of the SAT score. Animals underwent 162 trials per session, 81 of which were non-cue trials, while 27 cue trials were utilized with 500 ms, 50 ms, and 25 ms cue duration (total of 81 cue trials). Attention training (pSAT) occurred in the absence of the house light, but after mastery of the pSAT condition, the house light was introduced. The SAT score was calculated using the above formula consisting of hits at each cue duration and total false alarms.

Since this task utilizes two different types of stimuli to be discriminated, signal detection theory measures were calculated to thoroughly evaluate performance on this task [6,47,48,49,50,51]. First, the SAT score was calculated using the following formula (h −f)/(2(h + f) − (h + f)^2^), generating a value ranging from 1.0 (all responses on cued trials were hits, and all responses on non-cued trials were correct rejections) to −1.0 (all responses on cued trials were misses, and all responses on non-cued trials were false alarms; See Figure 10). It was determined that chance performance on this measure is 0 ± 0.17 [6]. Two additional measures derived from signal detection theory were utilized to determine both perceptual sensitivity (A′), as well as response bias (B″) [48,50,51]. A′ is a subject-specific measure of strength of signal relative to noise and is unaffected by response bias. A′ was calculated as [0.5 + ((h − f) (1 + h − f)/4h(1 − f))], producing a value ranging from 0.5 (subject cannot distinguish signal from background) to 1 (subject always registered a hit and never registered a false alarm). B″, on the other hand, is a measure of response bias calculated as ((h(1 − h) − f(1 − f)/(h(1 − h) +f(1 − f)), generating values ranging from 1.0 (respond that the cue was never present) and −1.0 (respond that the cue was always present).

### 3.4. ACh Grab 3.0 Viral Infusion Surgery and ACh 3.0 Recording

One week following mastery of the SAT task, subjects underwent stereotaxic surgery in preparation for in vivo fiber photometry recording. Rats were administered a mixture of dexmedetomidine (Dexdomitor, Zoetis, Kalamazoo, MI, USA; 0.04 mg/kg) and ketamine (Ketaset, Zoetis, Kalamazoo, MI, USA; 85 mg/kg) intraperitoneally for anesthesia. A genetically encoded calcium indicator for the detection of ACh activity (1 μL, AAV9-hSyn-ACh3.0; AddGene; Watertown, MA, USA) was infused into the medial prefrontal cortex (AP (2.7 mm); ML (±0.7 mm); DV (−3.0 mm) [52]), prior to fiber optic cannula placement (MFC_400/430-0.48_5mm_MF1.25_FLT, Doric Lenses, Quebec, QC, Canada), at the same AP and ML coordinates, but 0.1 mm dorsal to the site of viral infusion. At the end of surgery, each subject received an intraperitoneal injection of Antisedan (atipamezole HCl, 5 g/kg) to reverse the sedation. Subcutaneous injections of carprofen (5 g/kg, Zoetis, Kalamazoo, MI, USA) were used as an analgesic prior to surgery, 24 h post-surgery, and 48 h post-surgery. Rats were allowed to recover for 3 weeks prior to behavioral testing and fiber photometry.

Following viral infusion and cannulation, animals were re-trained to the pSAT and SAT tasks with the stated criteria. On the session following the third day of passing the SAT, a one-meter-long fiber optic patch cord (MFP_400/430/1100-0.57_1m_FC-MF1.25_LAF, Doric Lenses) was attached to the fiber optic cannula, and rats were left to habituate in the operant chamber for 30 min before collecting a baseline signal of AChGRAB 3.0. Following baseline collection, the SAT task was started with recording of AChGRAB 3.0. The ACh indicator was excited with a ~490 nm signal, and a second ~405 nm signal was used as a control. While the signal collected from the ~490 nm channel reflects the ACh binding to a GFP-conjugated nonfunctional M3 ACh receptor, the ~405 isosbestic control channel is used to correct for motion artifact and background fluorescence. Signals emitted from AChGRAB 3.0 were collected with the signal processor (RZ10x Processor) running Synapse software (version 3, Tucker Davis Technology) through the same fiber optic patch cord. Behavioral inputs were time-stamped onto the collected 490 nm and 405 nm channels via TTL pulses that marked cue light presentation, lever extension and lever pressing, and reward delivery. We were unable to collect fiber photometry data from 6 subjects due to loss of head cap prior to meeting passing criteria (one male CON, one female CON, two female AIE, two female CON-LM).

### 3.5. ACh 3.0 Data Analysis

All analyses for ACh GRAB 3.0 activity were conducted in Spyder 5.0.3 running a kernel of Python 3.4. the signal collected from the 490 nm channel was transformed to ΔF/F by subtracting fluorescence detected in the 490 nm channel from the 405 nm channel before dividing the result by the signal in the 405 nm channel. Peri-event histograms were produced by measuring the ΔF/F 2 s before and after each time-stamped behavioral event. Peri-event histograms were then plotted as a normalized z-score of the entire recording session. The Z-score of rats belonging to the same treatment group were then averaged at each behavioral event. To account for photobleaching over the course of the SAT task, only the first 20 min of ACh GRAB 3.0 activity was used for analyses. From the generated Z-score, peak Z-scores pertaining to behavioral events in cued trials (cue presentation preceding hits, cue presentation preceding misses, hits and misses) at each time interval were measured alongside the peak Z-score during correct rejections and false alarms of non-cued trials. Area under the curve was also calculated using sklearn.metrics.auc to measure the total fluorescent output recorded during each task epoch relative to the within-trial baseline period.

### 3.6. Tissue Collection

One week after the end of operant training, rats were sacrificed (Fatal-Plus, Vortech Pharmaceuticals, Dearborn, MI, USA), and transcardially perfused (Master Easy-Load Console Drive, 7518-00; Cole-Palmer Instruments, Vernon Hills, IL, USA) with cold phosphate buffer saline and cold 4% paraformaldehyde (PFA; Electron Microscopy Services, Hatfield, PA, USA). Tissue was then postfixed for 24 h in 4% PFA before transferring to a 30% sucrose solution in 0.1 M PBS and stored at 4 °C. Brains were sliced coronally on a freezing sliding microtome (Sm2000r; Lecia Biosystems, Wetzler, Germany) at 40 μm and then stored at −20 °C in cryoprotectant (62.8 mg NaH_2_HPO_4_, 160 mL dH_2_O, 120 mL ethylene glycol, and 120 mL glycerol). To confirm AChGRAB 3.0 viral and fiber optic cannula placement, mPFC tissue sections were immediately mounted onto gelatinized slides and cover slipped with Prolong Glass Antifade (Thermo Scientific, Waltham, MA, USA) and imaged at 10× using an Olympus VS200 Slide Scanner (Olympus, Breinigsville, PA, USA).

### 3.7. ChAT and TrkA Immunofluorescence

Four sections per subject (approximately −0.72, −1.08, −1.44, and −1.8AP) were used to investigate treatment effects of AIE and LM11A-31 on the expression of ChAT+/TrkA−, and ChAT+/TrkA+ cell types within the NbM. Free-floating sections were washed in 0.1 M Tris-buffered saline (TBS), followed by a 30 min quench process in 0.3% H_2_O_2_ in 0.1 M TBS. NbM sections were again washed in 0.1 M TBS before undergoing an antigen retrieval step where tissues were incubated in a sodium citrate buffer at 80 °C for 30 min. Sections were again washed in 0.1 M TBS prior to being placed in a blocking solution (4% normal donkey serum, 0.1% Triton X-100) for one hour. Tissues were then transferred to a ChAT primary and TrkA primary antibody solution with blocking buffer (1:200 dilution goat anti-ChAT polyclonal AB143, Millipore EMD, Billerica, MA, USA; 1:200 rabbit anti-TrkA polyclonal 06-574, Millipore EMD, Billerica, MA, USA) and incubated overnight at 4 °C. Following 3× 5 min washes in 0.1 M TBS, NBM sections were then incubated in blocking buffer with secondary antibody (1:300 dilution Alexa fluor 488 Donkey anti Rabbit A-21206, Thermo Scientific, Waltham, MA, USA; 1:400 dilution Alexa Fluor 680 Donkey anti Goat 705-625-147, Jackson Immuno Research, West Grove, PA, USA) for 2 h. Afterwards, sections were then washed in 0.1 M TBS before being cover slipped with Prolong Glass Antifade (Thermo Scientific, Waltham, MA, USA).

### 3.8. Microscopy and ChAT/TrkA Cell Counting

Slides were randomly coded and imaged at 20× using an Olympus VS200 Slide Scanner. Images were exported to FIJI through the Olympus plugin. The NIH Image J cell counting tool was used to quantify the total number of ChAT+/TrkA−, and ChAT+/TrkA+ cholinergic neuronal phenotypes in one hemisphere across all NbM sections. Since only one hemisphere was counted, the total number of ChAT+TrkA+ and ChAT+TrkA− cells were doubled.

## 4. Discussion

There are several hypotheses about the neural dysfunction associated with adolescent heavy alcohol exposure [2,53,54]. Recently, neurotrophin disruption has emerged as a key player in alcohol-related brain damage—particularly loss of the cholinergic phenotype following adolescent binge-type ethanol exposure [22,55]. Although modulating the p75NTR has been shown to be neuroprotective in aging and AD models, especially in basal forebrain cholinergic cell populations [41,43,44], no previous work has been undertaken to determine if targeting the p75NTR during binge ethanol exposure would protect cholinergic neuronal phenotypes and/or improve long-term neurocognitive outcomes. Here we show that LM11A-31 treatment during AIE protects attentional performance, cortical ACh activity, and basal forebrain cholinergic cell populations, regardless of sex. However, treatment with LM11A-31 in control adolescent female rats suppressed NbM cholinergic neurons. These results suggest that blocking the p75NTR during normal adolescence may produce adverse consequences in cholinergic phenotype expression.

The first goal of this study was to characterize the attentional performance of rats that had undergone AIE treatment. Given that AIE has been consistently shown to lead to loss of basal forebrain cholinergic phenotype expression and reductions in cortical ACh release [18,19,20,21,22,56], we expected that AIE would also negatively affect attentional performance, as ACh is a key driver of attention [6,7,8]. We found that AIE caused an impairment during SAT pretraining. However, co-treatment of LM11A-31 during AIE blocked the emergence of these deficits. We found AIE-induced impairments in both perceptual sensitivity (A′) and response bias (B″) during pSAT but not SAT. Rats exposed to AIE displayed reductions in A′ in rats exposed to AIE, suggesting that AIE decreases cue-detection ability. It appears that AIE treatment results in the failure to adopt a conservative approach to cue detection, evident with B″ being closer to 0; see [6,47,48,49,50,51]. Both decrements in response bias and perceptual sensitivity were prevented through LM11A-31 modulation during AIE treatment. AIE-treated rats, along with the CON-LM group, were more likely to indicate the presence of the cue regardless of whether the cue is presented.

Performance on the pSAT and SAT tasks also revealed a few interesting sex-specific effects. On average, female rats took longer to master the pSAT task and demonstrated increased latencies to lever press at shorter cue durations than male rats but not during 500 ms cue or no cue trials. While findings of sex differences in attention are mixed, males have been found to have greater vigilance, while females tend to have reductions in reaction time but greater inhibitory control [46,57,58,59,60,61,62,63]. Greater inhibitory control typically observed in females most likely contributed to the greater response bias observed in female rats compared to male rats [59,61,63]. In addition, female rats also demonstrated greater bottom-up attention than male rats as revealed through higher perceptual sensitivity scores on pSAT—which conflicts with previous findings in females [46,62,64]. Collectively, the differences between male and female rats in perceptual sensitivity and response bias also led to females having higher SAT scores, at each cue duration, compared to males.

Although differences between AIE and CON rats in sustained attention performance were not evident under the more taxing condition when the house light was on, it is possible that attention deficits in AIE-treated animals may have been masked by increases in task difficulty or a floor effect. We saw a drop in performance in all rats when rats were transferred to the SAT version, which is expected [46].

Previous research has shown that ChAT cell loss in the basal forebrain, induced by IgG Saporin lesions, leads to impairments on SAT [6]. The lack of AIE effect on the more complex SAT task could also be because AIE does not produce the same magnitude of cholinergic pathology (25% loss) typically observed following cholinergic lesion studies (~50% loss; [6,65]). Although sustained attention has not been examined in developmental models of ethanol exposure, studies in human adolescents have shown that a history of alcohol misuse is associated with worsening performance on sustained attention [66,67,68]. The findings from this study can extend the observed sustained attention deficits in human adolescents to rodents treated with the adolescent intermittent ethanol paradigm.

In animal models of adolescent binge drinking, previous work using the five-choice serial reaction time task (5-CSRTT) and attentional set shifting reveal mixed results pertaining to attentional deficits [20,69,70,71,72,73]. Previous work investigating differences in attentional performance following 2 g/kg ethanol exposure on a two-day-on two-day-off cycle from early adolescence (PND 30–45) in D2 and B6 male mice found that ethanol did not produce attentional impairments on the 5-CSRTT, while late-adolescent to early-adulthood treatment (PND 45–60) increased the number of premature responses [70]. Conversely, 5 g/kg ethanol exposure on a two-day-on two-day-off schedule from PND 25–57 in male Wistar rats had improved attentional performance on the 5-CSRTT, stemming from fewer omissions, than control rats [69]. Similarly, 5 g/kg ethanol treatment from PND 25–54 in Sprague Dawley rats has been shown to increase [72] and decrease attentional set-shifting performance [20,73]. The conflicting findings on attentional performance following AIE could be due to underlying differences in ethanol-exposure procedures or the various cognitive domains that affect performance on 5-CSRTT and ASST, namely impulsivity, cognitive flexibility, and behavioral flexibility. AIE-associated changes in Pavlovian associations towards reward-related cues most likely contribute to the differences in performance across these tasks. Pavlovian-conditioned approach (PCA) has shown that AIE predisposes animals to greater incentive salience toward reward-related cues (conditioned stimulus), termed sign tracking (ST), as opposed to goal tracking (GT), where attention is preferentially directed to the reward delivery area (unconditioned stimulus) [74,75]. While most investigations into sign tracking focus on greater vulnerability to substance use disorders and relapse, sign trackers have been shown to outperform goal trackers in set shifting [76] and in acquisition of 5-CSRTT [77] but not pSAT [46]. However, sign trackers appear to be more dependent on bottom-up attentional processing [46] which agrees with the findings of this study, where AIE-treated animals appear to have delayed development of top-down attentional control, despite impaired bottom-up attentional processing. Overall, to limit confounding differences that incentive salience has on task acquisition, future assessments of attention should incorporate PCA to control for the distribution of STs and GTs within treatment conditions.

Recording cholinergic activity in the mPFC via fiber photometry during SAT revealed that AIE-treated animals have blunted ACh responses to cue presentation and reward, and that LM11A-31 treatment during ethanol exposure had a protective effect on prefrontal cortical ACh signaling. Previous experiments in cortical ACh signaling following AIE have identified blunting of activity-related ACh release in the mPFC and the OFC through in vivo microdialysis during a spatial working-memory task [18,20]. However, microdialysis is temporally limited in its ability to record ACh and is unable to disentangle tonic and phasic ACh release. Previous work has shown that optogenetic stimulation of basal forebrain cholinergic neurons during cue presentation facilitates cue detection on SAT, while optogenetic inhibition of this cholinergic population inhibits SAT performance [7]. Moreover, using choline-sensitive microelectrodes, prefrontal cortical increases during cue detection [8] and cholinergic basal forebrain neuron activity increases in response to reward [12]. Interestingly, although AIE-treated animals did not differ in behavioral indices from CON- or LM11A-31-treated animals during the SAT task, differences in ACh activity, and loss of cholinergic neurons in the NbM persist. While reductions in PFC ACh activity were found in AIE-treated animals despite similar performance on SAT, prefrontal cortical reciprocal projections to the posterior parietal cortex and basal forebrain likely compensate for the reductions in ACh during cue detection. Here, the PFC can mediate top-down attentional control through PFC to posterior parietal cortex glutamatergic transmission [78].

AIE produced reductions in ChAT+ expression in male and female rats, as shown in previous studies [18,19,20,21,22]. Uniquely, we found that the co-expression of ChAT+TrkA+ was significantly reduced following treatment with AIE, but LM11A-31 treatment spared this population. Over 90% of cholinergic neurons in the basal forebrain have co-staining with TrkA [79], and these neurons display the greatest plasticity, over ChAT+TrkA−, to NGF [80]. The plasticity response to NGF is recovery of the cholinergic phenotype, increase in soma size, and neurite outgrowth following neurotoxicity [79,80]. By inhibiting the p75NTR during AIE, the basal forebrain cholinergic phenotype responsive to NGF cortical ACh release, and attentional performance were spared from AIE-induced impairments. Together, this indicates that the degeneration of cholinergic neurons in this model is in part mediated by proneurotrophin signaling at the p75NTR. Previous work found that loss of Trk receptors precedes degeneration of cholinergic neurons, and that cholinergic cell degeneration occurs through the p75NTR [81]. This supports our findings that blocking pro-neurotrophin p75NTR signaling during ethanol exposure protects cholinergic cell populations. Interestingly, LM11A-31 also prevented the loss of the ChAT+TrkA+ subpopulation indicating that cholinergic access to cortically derived neurotrophins was also preserved, as Trk expression positively correlates with neurotrophin signaling [82,83,84]. Whether AIE-induced reductions in TrkA expression in cholinergic neurons precede reductions in ChAT phenotype expression is unknown; however, restoration of the cholinergic phenotype also coincides with demethylation of cholinergic and TrkA promoters [56].

Interestingly, LM11A-31 treatment during adolescence in water-exposed females did lead to reductions in ChAT+TrkA+ and ChAT+TrkA− neurons in adulthood. This drug has been shown not to have toxic effects in normal adult rodents [39]. However, the selective effect of this drug in females during adolescence, alongside reductions in mPFC ACh signaling during reward, and corresponding attentional impairments in adulthood, suggest that inhibiting p75NTR activity during development in intact female animals or withdrawal from the LM11A-31 in early adulthood led to degeneration of cholinergic neurons. It is known that during development the p75NTR is critical to the promotion of Trk signaling [85]. Thus, inhibiting the p75NTR during normal adolescence may have a detrimental effect on the survival of ChAT neurons. Such deficits would likely affect successful aging in females, making the cholinergic and other systems that rely on it vulnerable to other disease states.

Taken together, these findings implicate AIE-associated changes in p75NTR-mediated signaling in driving losses of the cholinergic phenotype in the basal forebrain, reductions in cortical ACh release, and behavioral impairments that persist despite discontinued exposure to ethanol in adulthood. Whether inhibiting the p75NTR during ethanol exposure would also protect cholinergic neurons that project to the hippocampus is not known at this time, but it has been demonstrated that LM11A-31 is effective at restoring cholinergic neurites in an AD murine model [86]. However, in the AIE model, hippocampal cholinergic activity is not as affected as cortical activity [87]. Thus, future work should examine whether inhibiting the p75NTR is effective at recovering other alcohol-related brain damage. Modulation of the p75NTR pathway is an important avenue for translational impact: it appears to be an effective target for treating alcohol use disorders by suppressing drinking [38] and recovering cholinergic dysfunction.

## Data Availability

The data that support the findings of this study are available on request from the corresponding author, [LMS].
